# Deciphering osteoarthritis cartilage heterogeneity: gene expression-guided patient stratification and microbiome correlations

**DOI:** 10.3389/fmicb.2026.1852881

**Published:** 2026-08-10

**Authors:** Xiaohong Huang, Dongxu Zhu, Tianrui Wang, Xiaodong Lian, Tengbo Yu, Yingze Zhang

**Affiliations:** 1 Shandong Institute of Traumatic Orthopedics, Medical Research Center, The Affiliated Hospital of Qingdao University, Qingdao, China; 2 School of Medicine, Nankai University, Tianjin, China; 3 Knee Preservation Center, Department of Orthopedics, The Affiliated Hospital of Qingdao University, Qingdao, China; 4 Department of Orthopedics, The Third Hospital of Hebei Medical University, Shijiazhuang, China; 5 Department of Orthopedic Surgery, Qingdao Hospital, University of Health and Rehabilitation Sciences (Qingdao Municipal Hospital), Qingdao, China

**Keywords:** arthroplasty, cartilage microbiome, chondroprotective agent, osteoarthritis, precise medicine

## Abstract

**Background:**

Demand for arthroplasty due to osteoarthritis (OA) is rising with population aging and need for revision surgeries. Efforts to slow OA progression and prevent post-operative complications have been ineffective, likely due to inadequate identification of the primary disease-driving mechanisms underlying cartilage destruction. The aim of this study was to identify the primary disease-driving mechanisms underlying OA cartilage destruction.

**Methods:**

Patient stratification was performed using a Gaussian mixture model according to the expression profiles of 14 OA indicators in the cartilage collected from total or unicompartmental knee arthroplasty and verified by cartilage microbiome.

**Results:**

Six endotypes were identified from 122 OA cartilage samples. Regarding extracellular matrix remodeling, Cluster 1 had high COL1A1 and was dominated by the Pseudomonadaceae family, potentially associated with fibrosis. Cluster 2 showed high COMP transcription and low MMP13, with a high level of species *Acinetobacter johnsonii*, which may affect matrix regeneration. Cluster 5 had high ADAMTS4 and abundant Streptococcus agalactiae, highlighting cartilage degradation in OA progression and infection risk in arthroplasty. Inflammation-driven destruction, as indicated by high MCP1 and CCR2 levels, was pronounced in Cluster 4 patients, with nine key taxa correlated with NTRK1, suggesting their role in pain sensitization. Regarding mechanosensitivity, Cluster 3 featured high TRPV4 expression and a predominance of the Mycobacteriales order, suggesting a link between mechanosensitivity and aging. Cluster 6 had secondary high levels of CCR2 and PIEZO1, indicating mechanoflammation.

**Conclusion:**

This study unveiled OA cartilage heterogeneity and highlighted the potential of using replaced cartilage, which may offer valuable clues for joint health assessment and future research into arthroplasty prognosis.

## Graphical Abstract

**Figure:**
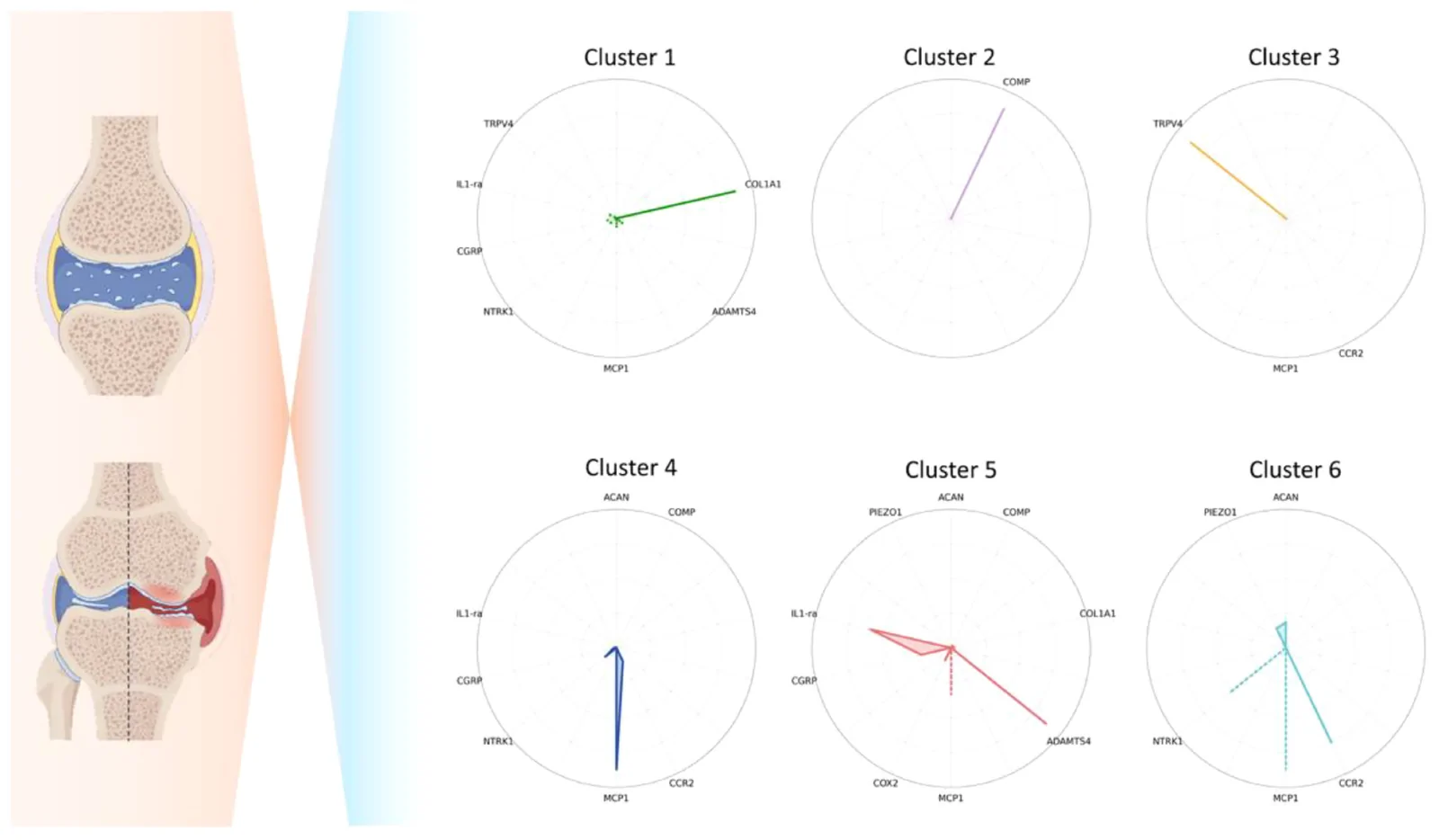
Six osteoarthritis (OA) cartilage endotypes were identified through Gaussian mixture model clustering based on the expression profiles of 14 OA-related indicators in cartilage samples from 122 knee arthroplasty patients.

## 1 Introduction

Osteoarthritis (OA), a heterogeneous degenerative disease, is a leading cause of disability and premature work loss (Hunter et al., 2020). The prevalence of symptomatic OA of either hand, or knee, or hip increases rapidly around age 50 and then levels off after age 70 (Oliveria et al., 1995). The demand for arthroplasty therefore increases with population aging, as well as with the need for revision surgeries. The incidence of revision total knee arthroplasty (TKA) is projected to increase by between 78% and 182% from 2014 to 2030 (Schwartz et al., 2020), partly attributing to post-operative complications such as infection and aseptic loosening of the prosthesis. Approximately 37% of patients who undergo unilateral TKA eventually require TKA for the contralateral knee within 10 years (Desouza and Shetty, 2025). And patients take nonsteroidal anti-inflammatory drugs (NSAIDs) or acetaminophen after unicompartmental knee arthroplasty (UKA) to alleviate contralateral compartment disorder (Tay et al., 2022). However, efforts, including anti-inflammatory agents, pain relievers and chondroprotective supplements, have been tried to slow down OA progression and prevent post-operative complications either failed or yielded unsatisfactory outcomes.

A meta-analysis indicates a significant discordance between radiographic OA and clinical symptoms: fewer than half of patients with radiographic OA report knee pain (ranging from 15% to 81%), while many with chronic pain show no radiographic signs (Bedson and Croft, 2008). Even in end-stage OA (Grade III/IV), joint microenvironments differ vastly. Some patients exhibit bone-on-bone contact and extensive osteophytes with minimal pain or inflammation. In contrast, others suffer severe redness, swelling, and burning pain, driven by persistent flare-ups of synovitis (Sellam and Berenbaum, 2010), the irritation of microcrystals (Fuerst et al., 2009), and systemic metabolic factors (Courties et al., 2017). Despite growing insights into the roles of inflammation, metabolic disorders, abnormal knee joint alignment, and traumatic events that drive primary OA (Tang et al., 2025), developing effective treatments for this idiopathic condition remains challenging. This may be attributable to inadequate identification of the primary disease-driving mechanisms underlying cartilage destruction in OA individuals.

This study employed a Gaussian mixture model (GMM) for patient stratification based on the expression profile of 14 OA indicators (listed in Supplementary Table 1) obtained from the replaced cartilage tissue. It has been reported that cartilage microbial DNA deposition influences local joint inflammation and OA progression (Dunn et al., 2020). Furthermore, the reliability of the clustering outcome was verified by cartilage microbiome, offering additional insights to OA etiology at the microbial level. This integrated strategy will furnish targeted data to guide effective therapy for contralateral components and other joints. The OA cartilage data, reflecting the local conditions of the replaced joint, could facilitate the development of targeted interventions aimed at mitigating postoperative complications and enhancing arthroplasty outcomes.

## 2 Methods

### 2.1 Participants and ethics

All participants provided informed consent. This study was approved by the institutional review board of the affiliated hospital of Qingdao university (No. QYFYEC2023-72) and conducted in accordance with the Declaration of Helsinki.

Participants aged 50 years and older diagnosed with primary OA based on their clinical manifestations and imageological examination, who met the indications for TKA or UKA and underwent surgery at the affiliated hospital of Qingdao university between July 2023 and July 2024, were enrolled. Exclusion criteria included arthroplasty due to specific types of arthritis (such as hemophilia, infection, autoimmune disease, rheumatoid arthritis, or congenital aplasia) and contraindications to the use of medications that may alter bone metabolism, including glucocorticoids, estrogens, calcium preparations, or others.

### 2.2 Demographic and medical records

Information on age, sex, body weight, and height was obtained from the medical records, and body mass index (BMI) was calculated. And posteroanterior and lateral X-ray projections were obtained under weight-bearing conditions to assess arthritis severity using the Kellgren–Lawrence (KL) grading scale. This radiographic evaluation was independently performed by two experienced radiologists.

### 2.3 Cartilage collection and sample preparation

The replaced joints were collected, including the medial and lateral compartments of the femoral distal end and tibial proximal end in TKA, as well as the medial compartment in UKA. The intact and eroded cartilage on the replaced tissue was dissected, rinsed with saline to remove any blood or other potential contaminants, collected in liquid nitrogen, and kept at −80 C°. The cartilage was cryogenically ground using a grinder mill (Spex CertiPrep, Middlesex, UK) in liquid nitrogen.

### 2.4 OA indicator expression profile

#### 2.4.1 OA indicator selection

Dysregulation of key extracellular matrix (ECM) components, such as aggrecan (ACAN), cartilage oligomeric matrix protein (COMP), and type II collagen alpha 1 (COL2A1), leads to alterations in cartilage volume and compromised texture. The expression of type I collagen alpha 1 (COL1A1) increases in late-stage OA cartilage where there is a shift toward a fibroblastic phenotype. In OA cartilage, degradative enzymes, such as matrix metalloproteinase 13 (MMP13) and a disintegrin and metalloproteinase with thrombospondin motifs 4 (ADAMTS4), primarily target the dysfunctional ECM components, facilitating cartilage remodeling and repair.

The inflammatory responses and cartilage destruction are closely intertwined. The monocyte chemoattractant protein 1 (MCP1)–C-C motif chemokine receptor 2 (CCR2) axis regulates the recruitment of inflammatory cells, exacerbating inflammation (Na et al., 2022). In contrast, cyclooxygenase-2 (COX2), via prostaglandin production (Sung et al., 2025), and interleukin-1 receptor antagonist (IL1-ra), by curbing IL-1’s inflammatory actions (Pathrikar et al., 2025), mitigate inflammation and pain.

In pain perception, calcitonin gene-related peptide (CGRP) acts as a pain transmitter, activating inflammatory cells and boosting MMP13 expression (Yang et al., 2024), and tropomyosin receptor kinase A (NTRK1) is involved in pain sensitization, steering neural differentiation and inflammatory responses that spur cartilage breakdown (O-Sullivan et al., 2022). Subsequently, transient receptor potential cation channel subfamily V member 4 (TRPV4) and piezo-type mechanosensitive ion channel component 1 (PIEZO1) detect and relay mechanical cues, thereby fueling pain perception, inflammatory reaction, and cartilage breakdown (Nims et al., 2024).

#### 2.4.2 RNA extraction and real-time PCR

The total RNA was extracted using Trizol (Ambion, USA). The concentration and purity of RNA was determined using a spectrophotometer (Nano-300, ALLSHENG, China). And cDNA was synthesized using the HiScript III RT SuperMix for qPCR (+gDNA wiper) (R323-01, Vazyme, China). The relative mRNA expression of target genes was quantified using the ChamQ Universal SYBR qPCR Master Mix (Q711-02, Vazyme) on an AriaMx Real-Time PCR System (Agilent Technologies, USA) with gene-specific primers (Supplementary Table 1). Glyceraldehyde-3-phosphate dehydrogenase (GAPDH) was served as a reference gene. Two technical replicates were performed.

#### 2.4.3 Data processing

The expression of indicators was centralized based on the widely recognized 2^–ΔΔCt^ method with minor modification (Livak and Schmittgen, 2001). Specifically, the difference between the Ct values of the target gene (Ct_*target*_) and the reference gene (Ct_*reference*_) was determined for each sample. These differences were then averaged across the cohort to determine a mean Ct difference (ΔCt). The ΔΔCt value was obtained by subtracting the overall mean from each individual difference. And the relative expression levels of indicators was calculated using the formula 2^–[ΔCt^ ^–ΔCt (mean)]^.

#### 2.4.4 Clustering

The data was log-transformed to account for skewed distribution. The processed indicator expression data was clustered using GMM with Python (version 9.3). This approach was specifically chosen because OA pathology typically exists on a continuous spectrum rather than in distinct, mutually exclusive stages. And unlike rigid hard-clustering methods, the probabilistic nature of GMM allows for soft clustering, which more accurately reflects the inherent biological continuity and overlapping characteristics of OA disease states. The optimum value for k was identifed based on the log-likelihood function, Akaike information criterion (AIC), Bayesian information criterion (BIC), and silhouette coefficient for clustering. And the average expression of indicators by clusters was revealed. Moreover, to specify the cluster difference, the eigenvalues were log-transformed to calculate the mean for each indicator, and the median indicator(s) of each cluster was unveiled using radar plot.

### 2.5 Microbiome

#### 2.5.1 2 bRAD-M sequencing and contamination control

2bRAD libraries were prepared according to the previous study (Zhou et al., 2024). The DNA extracted from the cartilage powder went through digesting, ligating, PCR, purifying, and sequencing in proper order. To ensure the authenticity of the microbial signals from the low-biomass cartilage environment, two types of negative controls were employed throughout the experimental process: (1) Extraction negatives (blank controls): these controls contained no sample material and were processed alongside the cartilage samples starting from the DNA extraction phase to monitor potential contamination from extraction reagents and labware; (2) Library preparation negatives (no-template controls): sterile water was used instead of DNA template during the library construction phase to identify any contaminants introduced during the PCR or ligation steps.

Raw sequencing data were in FASTQ format. Notably, the sequence yields from the negative controls were several orders of magnitude lower than those from the cartilage samples: extraction negatives yielded 2,000 to 130,000 reads, while library preparation negatives yielded only 20 to 3,000 reads. Given this significant disparity in sequencing depth and the minimal impact of these low-frequency sequences on the overall microbial community structure, the raw reads were proceeded to subsequent analysis. To further minimize potential contaminant effects, taxa present in fewer than 10% of all cartilage samples were excluded from subsequent analyses. Paired-end reads were then preprocessed using divisive amplicon denoising algorithm 2 (DADA2) with the default parameters of quantitative insights into microbial ecology version 2 (QIIME2) to detect and cut off the adapter. The representative read of each amplicon sequence variant (ASV) was selected using QIIME 2 package. All representative reads were annotated and blasted against Silva database version 138.

#### 2.5.2 Bioinformatic analysis

The α-diversity was estimated using the Chao1, Shannon, and Simpson indexes. The β-diversity was assessed through principal coordinates analysis (PCoA), and the significance of differences in microbial composition among clusters was determined using permutational multivariate analysis of variance (PERMANOVA). Linear discriminant analysis (LDA) effect size (LEfSe) was used to identify the differential taxa among groups, with an LDA score threshold of 3.0. And the relative abundance of these taxa was unveiled. Kyoto Encyclopedia of Genes and Genomes (KEGG) analysis were applied for function predition. The cluster-specific KEGG L3 pathways were determined on the base of these significant pathways.

#### 2.5.3 Spearman correlation

To explore the correlations between the taxa highlighted in LEfSe and OA indicator expression, centered log-ratio (CLR) transformation was applied to the microbial abundance data. Concurrently, log transformation and variance stabilizing transformation (VST) were used to normalize the gene expression data. Spearman correlation coefficients were calculated to assess the relationships between taxa and genes. To control for multiple testing, Benjamini–Hochberg false discovery rate (FDR) correction was applied across all tested pairs, with a significance threshold of *q* < 0.05. The results were refined to include only those pairs exhibiting an absolute correlation coefficient (|ρ|) of at least 0.2 and an FDR-adjusted *q*-value below 0.05. A taxon–gene network was then constructed using the NetworkX library.

### 2.6 Statistical assessment of cluster differences

The differences in numerical variables regarding the demographic and clinic characteristics, gene expression and microbial contents among clusters were assessed using Kruskal–Wallis H test. To manage the false discovery rate, *post-hoc p*-values obtained from Mann–Whitney U test were adjusted using Benjamini–Hochberg correction. Additionally, global Chi-square tests were performed to evaluate the overall association between categorical variables and clustering results. A *p*-value < 0.05 was considered statistically significant.

## 3 Results

### 3.1 Baseline characteristics of the cohort

A total of 122 patients were recruited in the cohort, comprising 28.69% males and 71.31% females, with a mean BMI of 25.333 kg/m^2^ (Table 1). Among them, 44.26% were at grade III and 55.74% at grade IV; half underwent TKA and half underwent UKA (Table 1).

**TABLE 1:** Demographic and clinical characteristics of the study participants.

Characteristics		Participants (*n* = 122)	Cluster 1 (*n* = 29)	Cluster 2 (*n* = 38)	Cluster 3 (*n* = 17)	Cluster 4 (*n* = 4)	Cluster 5 (*n* = 14)	Cluster 6 (*n* = 20)	*P*-value
Age (y)^a^		67.000 (63.000, 73.000)	68.000 (63.000, 73.000)	70.000 (62.250, 75.500)	71.000 (64.000, 71.000)	72.500 (69.250, 74.250)	65.000 (64.000, 70.000)	66.000 (62.750, 72.750)	0.812
Sex^b^	Male	28.689%	24.138%	28.947%	11.765%	0.000%	50.000%	40.000%	0.124
	Female	71.311%	75.862%	71.053%	88.235%	100.000%	50.000%	60.000%	
Height (cm)^a^		160.000 (156.250, 167.000)	160.000 (155.000, 168.000)	160.000 (157.250, 166.500)	160.000 (158.000, 165.000)	160.000 (153.750, 166.750)	160.000 (158.500, 167.500)	161.000 (158.000, 167.250)	0.937
Weight (kg)^a^		66.000 (60.000, 75.000)	68.000 (60.000, 75.000)	65.000 (59.250, 74.500)	66.000 (57.000, 72.500)	59.000 (57.250, 69.500)	66.000 (60.625, 76.500)	70.000 (62.750, 75.000)	0.690
BMI (kg/cm^2^)^a^		25.333 (23.401, 28.339)	26.730 (23.661, 28.345)	25.128 (23.259, 28.116)	25.149 (22.507, 27.380)	24.335 (22.679, 27.615)	25.781 (23.118, 28.063)	25.313 (24.131, 28.921)	0.784
KL grading^b^	III	44.262%	37.931%	55.263%	47.059%	100.000%	28.571%	50.000%	0.204
	IV	55.738%	62.069%	44.737%	52.941%	0.000%	71.429%	50.000%	
Knee arthroplasty^b^	TKA	52.459%	58.621%	50.000%	52.941%	25.000%	42.857%	60.000%	0.743
	UKA	47.541%	41.379%	50.000%	47.059%	75.000%	57.143%	40.000%	

*^a^*Data are reported as median (Q1, Q3).

*^b^*Data are reported as percentages.

### 3.2 Clustering stability and cluster property

The optimum value for k (number of clusters) was determined via comprehensively evaluating the model fit statistics (Supplementary Table 2). The clustering analysis revealed that the highest quality clusters were achieved when k = 6 and k = 7. However, when k = 7, the radar plot showed that Cluster 4 and Cluster 7 shared similar features, which somewhat diminished the distinctiveness of the clustering (Supplementary Figure 1).

**FIGURE 1:**
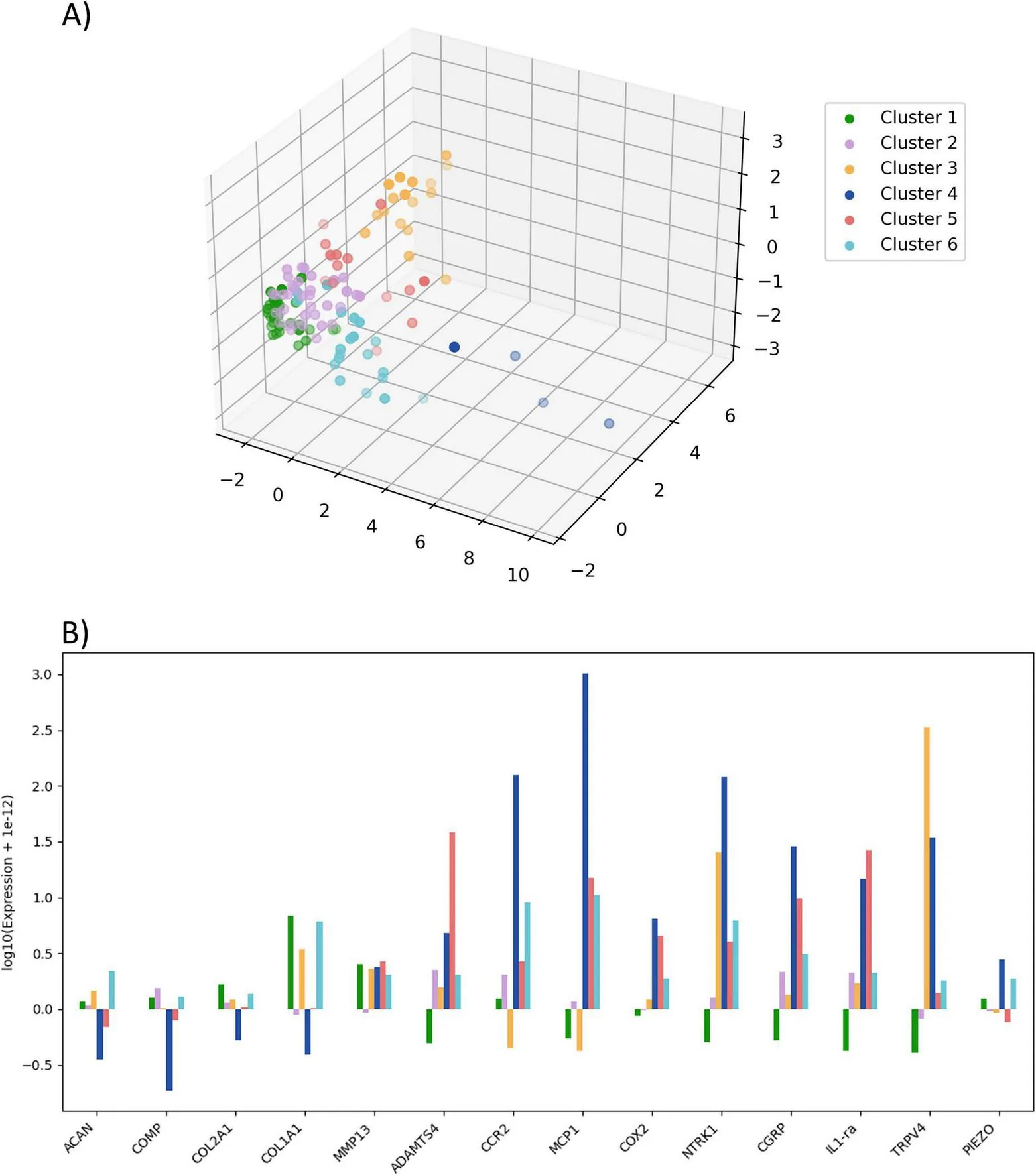
Clustering of OA cartilage samples and gene expression profile of clusters. **(A)** 3D Principal Component Analysis (PCA) plot demonstrating the spatial separation of cartilage samples. The samples are categorized into six distinct clusters using the Gaussian Mixture Model (GMM) based on the expression levels of 14 Osteoarthritis (OA) indicators. **(B)** Comparative expression profiles of the 14 OA indicators across clusters.

**FIGURE 2:**
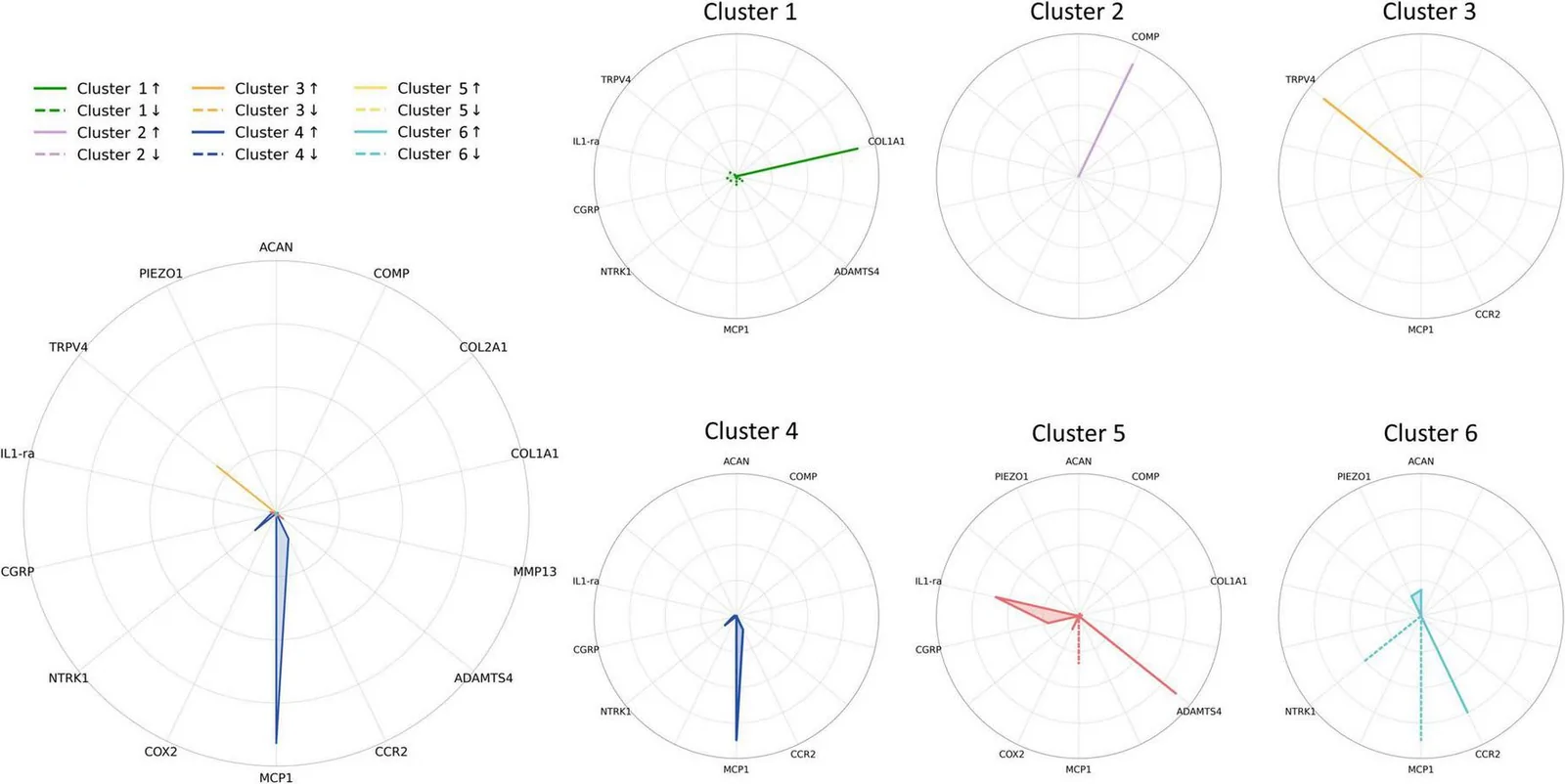
Signature OA indicators across cartilage clusters. Individual radar plots display the median expression profiles of 14 OA-related indicators for each cluster (Cluster 1–6), i.e., the corresponding indicator has been highlighted in the subgraph when its expression exhibited significant alteration compared to other clusters. The full line denote significantly up-regulated indicators, while the dotted line represent significantly down-regulated indicators. Subgraph radius is automatically adapted to the maximum value of the correspoding cluster(s).

When k = 6, the 14 OA indicators exhibited distinct expression patterns across the six clusters (Figures 1A, B). Despite the demographic and clinical characteristics of clusters were similar (Table 1), the cluster-specific biomarker expression patterns were distinct (Figure 2). Specifically:

◼ Cluster 1 patients had the highest level of COL1A1 and the lowest level of ADAMTS4, TRPV4, NTRK1, CGRP and IL1-ra, as well as a lower level of MCP1.
◼ Cluster 2 patients showed the highest transcription of COMP.
◼ Cluster 3 had the highest TRPV4 exrpession as well as the lowest MCP1 and CCR2 expression.
◼ Cluster 4 exhibited extremely high levels of MCP1, CCR2, CGRP and NTRK1 and the lowest ACAN and COMP expression, as well as a higher level of IL1-ra.
◼ Cluster 5 had highest levels of ADAMTS4 and IL1-ra, the lowest expression of PIEZO1, and the second lowest level of MCP1, CGRP, COX2 ACAN, COMP and COL1A1 after Cluster 4.
◼ Cluster 6 had the highest ACAN expression and the secondary highest levels of CCR2 and PIEZO1.

### 3.3 Microbial characteristics of clusters

**FIGURE 3:**
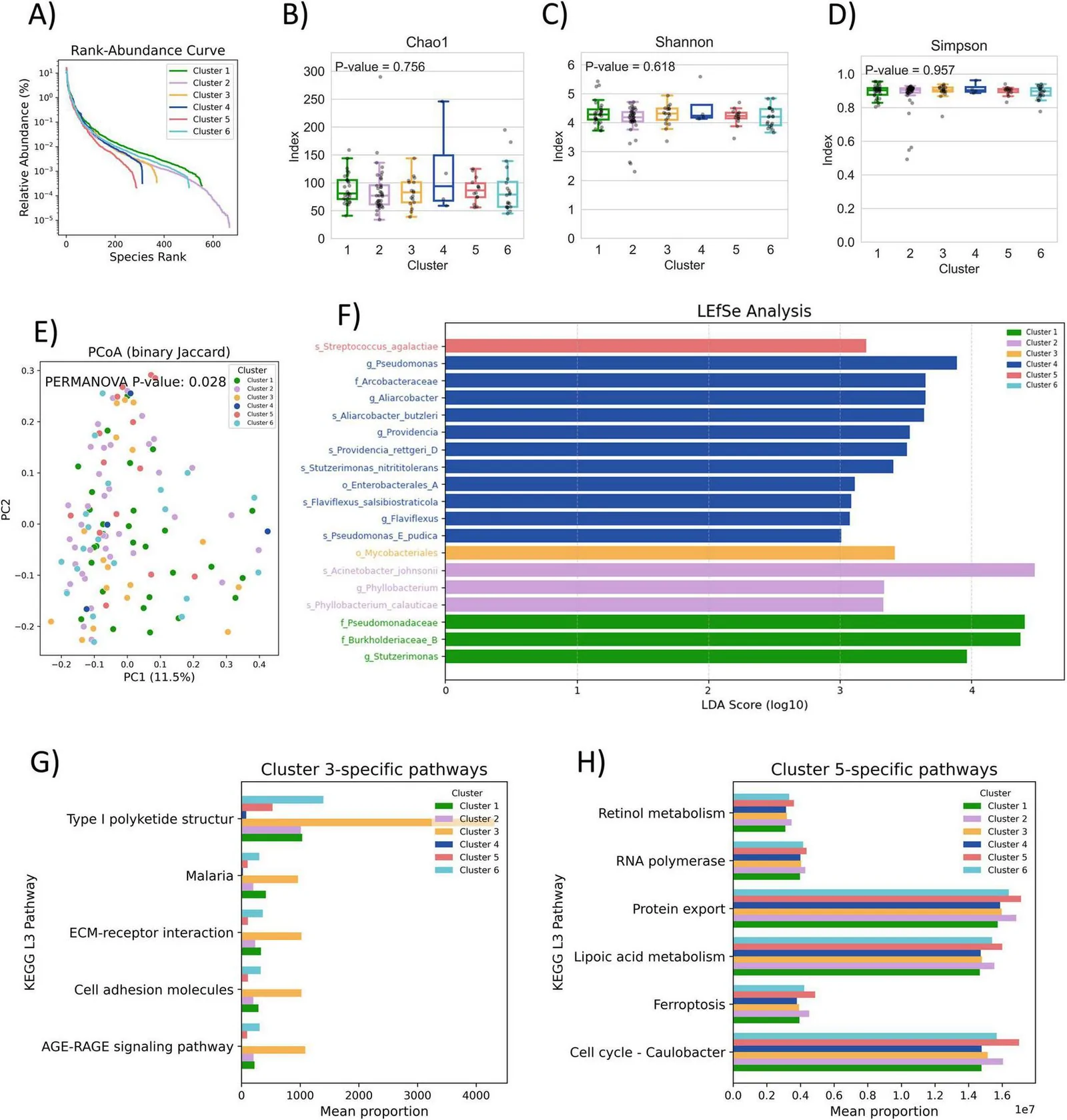
Microbiotic profile of clusters. **(A)** Rank-abundance curve illustrating the species richness and evenness within each cluster. **(B–D)** The α-diversity indices, including Chao1, Shannon, and Simpson. **(E)** The β-diversity visualized by Principal Coordinate Analysis (PCoA) based on binary Jaccard distance. The PERMANOVA *P*-value (*P* = 0.028) indicates significant differences in microbial community structure between clusters. **(F)** Linear discriminant analysis Effect Size (LEfSe) identifying discriminatory taxa (biomarkers) with LDA scores > 3.0. **(G, H)** Functional prediction of microbial communities. Significant KEGG Level 3 (L3) pathways specifically enriched in Cluster 3 and Cluster 5, respectively.

The confidenece of clusters has been verified by the distinct microbial taxonomic composition and distribution among clusters (Figure 3E and Supplementary Figure 2). Although Cluster 1 ranked highest in terms of the relative abundance and Cluster 5 ranked the lowest (Figure 3A), the α-diversity of clusters was not significant (Figures 3B–D).

**FIGURE 4:**
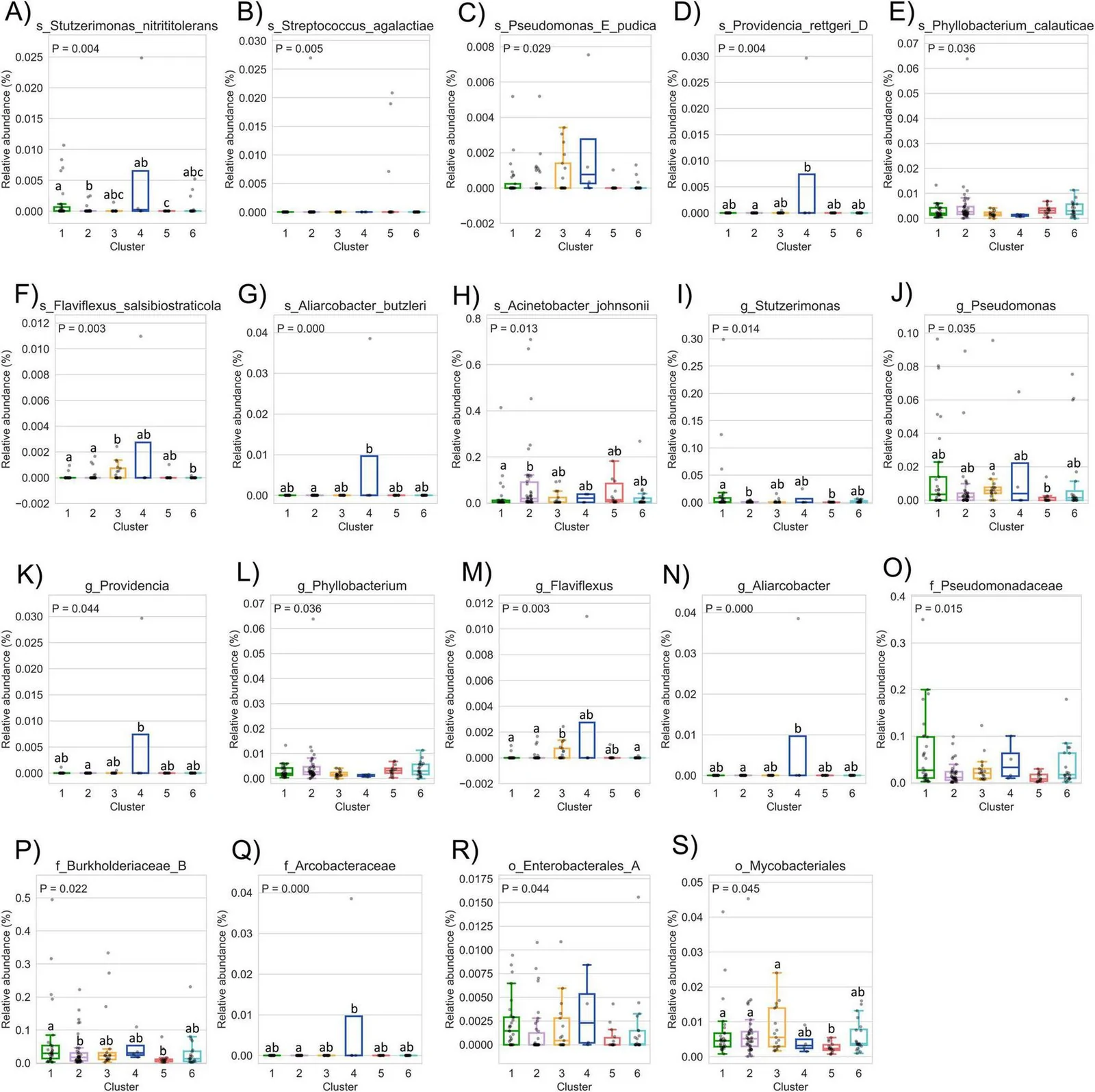
Relative abundance of discriminatory microbial taxa identified by LEfSe analysis across cartilage clusters. **(A–H)** Species level: Stutzerimonas nitrititolerans, Streptococcus agalactiae, Pseudomonas E pudica, Providencia rettgeri D, Phyllobacterium calauticae, Flaviflexus salsibiostaticola, Aliarcobacter butzleri, and Acinetobacter johnsonii. **(I–N)** Genus level: Stutzerimonas, Pseudomonas, Providencia, Phyllobacterium, Flaviflexus, and Aliarcobacter. **(O–Q)** Family level: Pseudomonadaceae, Burkholderiaceae B, and Arcobacteraceae. **(R, S)** Order level: Enterobacterales A and Mycobacteriales. The a, b superscripts indicate a statistical significance among clusters. The box-plot shows the median (line) and 25–75% percentiles (whiskers).

The key taxa that contributed to the cluster difference were identified using LEfSe (Figure 3F and Supplementary Table 3), and the relative abundance of these taxa was highlighted (Figure 4). The LDA score analysis revealed three key taxa in Cluster 1, including the families *Burkholderiaceae_B* and *Pseudomonadaceae* and the genus *Stutzerimonas*, which is a member of the *Pseudomonadaceae* family. Notably, the relative abundance of the genus *Stutzerimonas* was significantly higher in Cluster 1 than in Clusters 2 and 5. In contrast, the dominant microbiota in Cluster 2 included the genus *Phyllobacterium* and the species *Phyllobacterium calauticae*, which is a member of the genus *Phyllobacterium*, as well as the species *Acinetobacter johnsonii*. The relative abundance of *Acinetobacter johnsonii* was significantly higher in Cluster 2 compared to Cluster 1. The order *Mycobacteriales* was found to be dominant in Cluster 3, exhibiting higher abundance in Clusters 1, 2, and 3 compared to Cluster 5. Cluster 4 had 11 key taxa, such as the family *Arcobacteraceae*, genera *Aliarcobacter*, *Flaviflexus*, and *Providencia*, and speices *Aliarcobacter butzleri*, *Flaviflexus salsibiostraticola*, *Providencia rettgeri D*, *Pseudomonas E pudica*, and *Stutzerimonas nitrititolerans*. Cluster 5 was dominant with the species *Streptococcus_agalactiae*, and no dominant taxon has been detected in the cartilage from Cluster 6 patients.

To elucidate the role of microbiota in the OA cartilage, significant KEGG L3 pathways were identified (Supplementary Table 4), with Cluster 3-specific and Cluster 5-specific pathways pinpointed, while the remaining clusters lacked distinctive pathways (Figures 3F, G and Supplementary Table 5). Notably, the activity of pathways such as AGE-RAGE signaling, cell adhesion molecules and ECM-receptor interaction were more pronounced in Cluster 3 than in other clusters, especially when comparing to Cluster 4. Meanwhile, Cluster 5 exhibited heightened activity in pathways related to the cell cycle-Caulobacter, ferroptosis, lipoic acid metabolism, protein export, RNA polymerase, and retinol metabolism.

### 3.4 Genus–gene network

**FIGURE 5:**
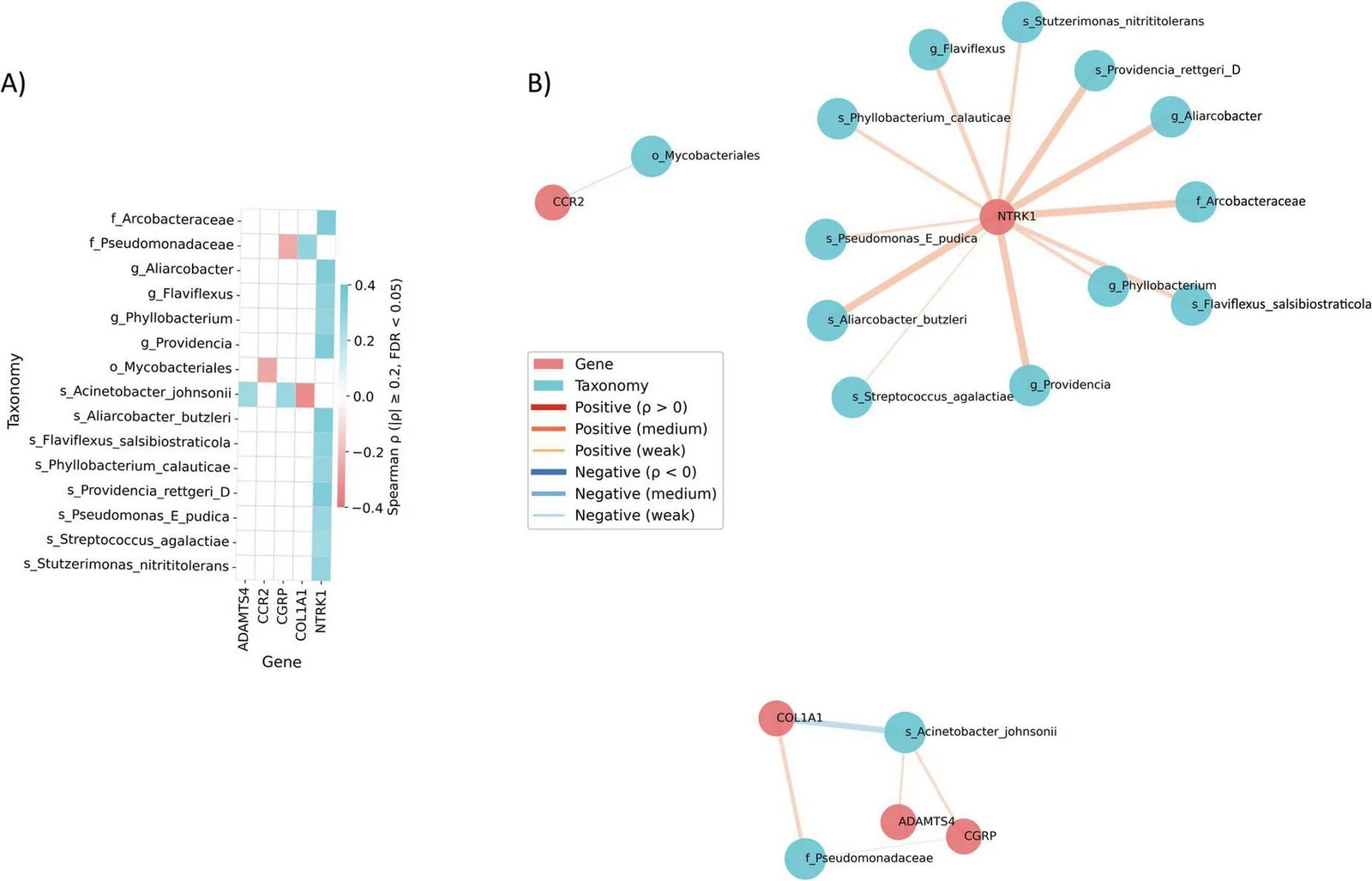
Taxon–gene network in the cartilage. **(A)** Heatmap showing Spearman correlation coefficients between LEfSe-identified differentially abundant taxa and OA-related gene expression. Microbial data were normalized via CLR transformation, while gene expression data underwent VST and log transformation. The color scale represents correlation strength. Only FDR-significant associations (*q* < 0.05, | ρ | ≥ 0.2) are displayed. **(B)** Bipartite network of taxon–gene interactions. Red nodes represent host genes, and blue nodes represent microbial taxa. Edge thickness is now proportional to | ρ | (thicker = stronger), enabling immediate visual assessment of correlation magnitude. Edge color indicates direction (red = positive, blue = negative).

To elucidate the role of microbiota in the OA endotype, we investigated the correlation between microbial content and OA indicator expression in the cartilage (Figure 5A), with significant taxon–gene pairs highlighted (Figure 5B and Supplementary Table 6). Specifically, the content of the species *Acinetobacter johnsonii* was negatively correlated with that of COL1A1. In contrast, the content of the family *Pseudomonadaceae* was negatively correlated with the expression of CCR2, but positively associated with that of CGRP. Moreover, the content of the order *Mycobacteriales* was negatively associated with the expression of CCR2. The NTRK1 expression was associated with the content of 12 taxa, including the families *Arcobacteraceae*, genera *Aliarcobacter*, *Flaviflexus*, *Phyllobacterium*, and *Providencia*, and species *Aliarcobacter butzleri*, *Flaviflexus salsibiostraticola*, *Phyllobacterium calauticae, Providencia rettgeri D*, *Pseudomonas E pudica*, *Streptococcus agalactiae*, and *Stutzerimonas nitrititolerans*.

## 4 Discussion

A combination of factors, such as the severity of joint damage, pain and functional limitations, comorbidities, and response to conservative treatments, contribute to the variability in individual responses and decisions regarding joint replacement. This study identified six endotypes of OA cartilage destruction based on OA indicator expression and cartilage microbiome. These findings provide a basis for exploring strategies to slow OA progression of other joint and may help address complications following arthroplasty, taking into account the diverse pathological aspects of OA cartilage destruction.

◼ ***Aberrant ECM regenration undermines tissue remodeling***

Cluster 1 patients exhibit a phenotype of highly fibrotic cartilage, accompainied by a diminished inflammatory response, lower pain perception, and reduced ECM degradation. And the microbiome of Cluster 1 cartilage was specifically dominated by the family *Pseudomonadaceaen* and the genus *Stutzerimonas*. This is consistent with the previous study that, comparing to normal cartilage, *Pseudomonadaceae* is increased in the hip-eroded cartilage (Dunn et al., 2020). which is suggested to its role in OA development and progression. Moreover, the identification of the *Pseudomonadaceae* family, including known pathogens like *Pseudomonas aeruginosa*, within the cartilage suggests that these microbes were already present in Cluster 1 patients prior to surgery. This pre-existing colonization highlights the importance of further investigating whether these latent microbial populations may influence perioperative outcomes (Ali et al., 2025). The content of *Pseudomonadaceae* was positively correlated with that of COL1A1 while negatively correlated with that of CGRP, which hints at the potential role of *Pseudomonadaceae* in articular cartilage fibrosis and pain perception. This pattern may indicate that lower pain perception could contribute to increased cartilage wear. Interventions such as knee function and muscle training, along with cartilage protectants like glucosamine and chondroitin, might offer theoretical benefits in protecting cartilage and slowing fibrosis, although this remains speculative based on the current data.

It has been reported that, in post-traumatic OA, the expression of both COL1 and COMP is elevated, resulting in a stiffer and more brittle regenerated cartilage tissue (Sebastian et al., 2020). However, in Cluster 2 patients, an uniquely high level of COMP, low MMP13 expression, and high abundance of the species *Acinetobacter johnsonii* has been characterized, rather than COL1. COMP facilitates the assembly of the ECM and stabilizes growth factors, and the serum COMP levels are correlated with radiological grading of OA (Verma and Dalal, 2013). The taxon–gene network involves COL1A1, CGRP and ADAMTS4, as well as the species *Acinetobacter johnsoni* and the family *Pseudomonadaceae*. *Acinetobacter johnsonii* exhibits a pattern that contrasts with the family *Pseudomonadaceae*, suggesting that these taxa may exert complementary roles on cartilage matrix remodeling. The differential COL1 and COMP expression patterns between Cluster 1 and 2 cartilage samples may be associated with differential cartilage repair and pain signal transmission in the development of OA. Moreover, *Acinetobacter spp*. form biofilms and enhance resistance to antibiotic treatment (Kim and Lim, 2023). This raises the hypothesis that the high abundance of *Acinetobacter johnsonii* in Cluster 2 cartilage could influence periprosthetic infection susceptibility, although this possibility awaits validation in longitudinal clinical cohorts.

◼ ***Overwhelming cartilage degradation hinders tissue repair***

MMP13 degrades collagen II and drives inflammation, while ADAMTS4 targets ACAN (Wang et al., 2023). The highest ADAMTS4 expression suggests the extensive degradation of Cluster 5 cartilage. Therefore, whether highly selective ADAMTS4 inhibitors, such as certain acetylurea compounds (Rossello et al., 2009), could delay or prevent cartilage destruction in the contralateral compartments of Cluster 5 patients remains a hypothesis that requires testing in preclinical models and future clinical trials. Moreover, Cluster 5 cartilage may have a higher microbial activity compared to other clusters, as evidenced by the highest activity in cell cycle-Caulobacter, protein export, and RNA polymerase activity, despite the lowest relative microbial abundance. It has been reported that retinoic acid exacerbates cartilage degradation and inflammation (Kamalathevan et al., 2021). The elevated level of retinoic acid induced by the altered microbial community in the cartilage potentially worsens OA, which thereby is paralleled with the highest ADAMTS4 expression in the Cluster 5 cartilage. Moreover, α-lipoic acid, a natural compound extensively found in human tissues, possesses antioxidant properties and exerts an anti-ferroptotic role on OA cartilage (Xu et al., 2023), whose increase is consistent with the high level of the chondroprotective IL1-ra in Cluster 5. Furthermore, the species *Streptococcus agalactiae* is a known pathogen of prosthetic joint infection, which may hematogenously disseminate to the prosthetic site via bacteremia, correlated with osteolysis and bone destruction (Tai et al., 2022). This observation raises the hypothesis that the presence of species *Streptococcus agalactiae* in Cluster 5 cartilage could be linked to an elevated risk of prosthetic loosening.

◼ ***Inflammation exacerbates OA progression and pain***

Extremely high levels of inflammatory mediators and pain preceptors have been identified in Cluster 4. By establishing a potent chemokine gradient, the MCP-1/CCR2 axis directs the active recruitment of monocytes and T cells from the joint space into the synovium and cartilage, thereby facilitating the transition from acute to chronic inflammation and subsequent cartilage degradation (Abbasifard and Khorramdelazad, 2024). Intra-articular injection of sCCR2 E3, a fusion protein encoding 20 amino acids of the E3 domain of CCR2, has been found to alleviate joint inflammation and cartilage damage in the rat OA model (Na et al., 2022). These findings suggest the hypothesis that targeting the MCP1–CCR2 axis could confer therapeutic benefits in Cluster 4 patients.

Multiple taxa represented Cluster 4, and the content of nine of the 11 taxa exhibited positive correlation with NTRK1, which is responsible for modulating pain sensitization (O-Sullivan et al., 2022). It hints that inflammation-linked neurovascular ingrowth and microbial deposition in OA cartilage may represent a potential basis for the increased pain perception observed in Cluster 4 patients. Targeting the nerve growth factor (NGF)–NTRK1 signaling transduction, Tanezumab an NGF antibody is associated with the risk of rapidly progressive OA (Dietz et al., 2021). These observations collectively suggest a hypothesis that microbial taxa may be involved in pain sensitization pathways, although the causal relationships and therapeutic implications remain to be elucidated.

◼ ***Elevated mechanosensitivity intensifies OA***

Cluster 3 cartilage exhibited the highest TRPV4 expression alongside the lowest levels of inflammatory indicators, and a pronounced abundance of the order *Mycobacteriales*. It has been hypothesized that chondrocytes in localized regions become hypersensitive to mechanical stimuli, leading to the activation of TRPV4 under conditions of low mechanical loading and osmotic alterations, resulting from uneven articular cartilage wear and inhomogeneous synovial fluid distribution (O’Conor et al., 2014). The expression of TRPV4 in the knee cartilage of OA patients is higher than that in normal cartilage, and conditional knockout of TRPV4 in the cartilage of adult mice alleviates age-related OA (O’Conor et al., 2016). These findings are consistent with the elevated activity of aging-related pathways observed from the Cluster 3 cartilage microbiome. And the order *Mycobacteriales*, which is dominant in Cluster 3 cartilage, has been reported to be associated with aging after spaceflight (Sun et al., 2025). Hence, these observations raise the hypothesis that senotherapeutic drugs may modulate aging-associated pathways in Cluster 3 cartilage. However, this conjecture requires validation using senescence-specific biomarkers (e.g., p16^INK4a^, p21, SA-β-gal, telomere length) in preclinical OA models and prospective clinical studies.

TRPV4 mediates mechanotransduction under physiological loading, while PIEZO1 primarily senses injurious mechanical tensile strain (Gao et al., 2022). Moreover, ACAN, which ensures elasticity and compression resistance in cartilage, is reduced in OA. This increased expression of ACAN may indicate some retained mobility in Cluster 6 patients compared to patients in other clusters. However, joint deformities, muscle strength imbalances, and daily activities may subject certain cartilage areas to excessive mechanical stress and activate PIEZO1, which in turn elevates CCR2 expression via mechanoflammation (Abbasifard and Khorramdelazad, 2024). Whether these mechanoflammatory processes could be counteracted by tailored physical exercise in Cluster 6 patients warrants further investigation.

***Future directions and limitations***

The main limitations of this study include the relatively small sample size and the cross-sectional design. Nevertheless, as longitudinal data from the planned multi-center validation cohort accumulate, future work will be able to assess whether these endotypes predict clinical outcomes and whether the proposed therapeutic strategies confer tangible benefits. This study probed the heterogeneity of OA cartilage destruction based on OA indicator expression profiles and microbiome data, underscoring the research value of replaced cartilage tissue. An external cohort to validate biomarker robustness may not be necessary here, as this study utilized established OA indicators to explore distinct endotypes of cartilage destruction, rather than applying machine learning to discover new diagnostic markers. Finally, applying a more stringent threshold of |ρ| ≥ 0.4 yielded no significant taxon-gene pairs, and even at |ρ| ≥ 0.3 only eight pairs remained (Supplementary Table 7). The inherently low-biomass nature of the cartilage microenvironment may preclude strong linear correlations between microbial DNA and host gene expression. Future studies with larger cohorts and integrated multi-omics approaches may help identify stronger associations. Moreover, the taxon–gene correlations and the proposed aging-related mechanism are hypothesis-generating and require direct experimental validation, e.g., *in vitro* chondrocyte functional assays and senescence marker measurement. Although the collected cartilage was rinsed with saline to remove blood and contaminants, directly localizing the microbes within the cartilage would further substantiate our findings.

Notably, the all-female composition and exclusive KL grade III status of Cluster 4 may at first raise concern for selection bias; however, we interpret these features as biologically consistent with the inflammatory OA phenotype. Female sex is a well-established risk factor for heightened synovitis, lower pain thresholds, and elevated inflammatory signaling in OA, and the fact that these patients underwent arthroplasty at KL III (rather than end-stage KL IV) suggests that intractable inflammatory pain – rather than mechanical wear – was the overriding indication for surgery in this subgroup, which aligns with the extreme elevation of MCP1, CCR2, CGRP, and NTRK1 observed in their cartilage. The GMM with multiple fit statistics and microbiome-based validation provided strong internal support for our clustering. However, an independent cohort is still needed to externally validate the reproducibility of the 6-cluster classification, particularly to determine whether the female predominance and exclusive KL III status observed in Cluster 4 represent true biological associations or cohort-specific characteristics.

## 5 Conclusions

Based on the gene expression of OA indicators and microbiomic profile of the cartilage tissue obtained from TKA or UKA, six endotypes of OA cartilage destruction have been identified in this study. These findings suggest the heterogeneity underlying OA cartilage destruction and underscore the potential of using replaced cartilage in joint health prediction and arthroplasty prognosis.

## Data Availability

The datasets presented in this study can be found in online repositories. The name of the repository and accession number can be found below: Sequence Read Archive (SRA) of the National Center for Biotechnology Information (NCBI) under BioProject accession number PRJNA1328927.
