# Supplementary material for: Deciphering osteoarthritis cartilage heterogeneity: gene expression-guided patient stratification and microbiome correlations
Source: Front Microbiol. 2026 Aug 10;17:1852881. doi: 10.3389/fmicb.2026.1852881 (PMC13500613; doi:10.3389/fmicb.2026.1852881)
Supplement: Supplementary file 1 [file Supplementary_file_1.docx]

**Supplemental files**

**Figure S1.** Clustering and median indicators of each cluster. A) 3D PCA of 14 indicators with GMM clustering (k = 7). B) The median indicator of the corresponding clusters.

**Figure S2.** Microbial composition of clusters. The top 30 genera demonstrate distinct differences in the relative abundance among clusters.

**Table S1.** Primers of the biomarkers and reference gene in qPCR.

| **Gene** | **Sequence** |
| --- | --- |
| ACAN (Mak, To, Fekir, Brooks, & Khan, 2022) | F: 5′-AGGGCGAGTGGAATGATGTT-3′ |
|  | R: 5′-GGTGGCTGTGCCCTTTTTAC-3′ |
| COMP (Koelling, Clauditz, Kaste, & Miosge, 2006) | F: 5'-AGGGAGATCGTG CAGACAA-3' |
|  | R: 5'-AGCTGGAGCTGTCCTGGTAG-3' |
| COL2A1 (Shi et al., 2019) | F: 5'-TCTACCCCAATCCAGCAAAC-3' |
|  | R: 5'-TGTTCTGGGAGCCTTCCGT-3' |
| COL1A1 (Neybecker et al., 2018) | F: 5′-AGGTGCTGATGGCTCTCCT-3′ |
|  | R: 5′-GGACCACTTTCACCCTTGT-3′ |
| MMP13 (Zuncheddu et al., 2022) | F: 5'-CGGCCACTCCTTAGGTCTTG-3' |
|  | R: 5'-TTTTGCCGGTGTAGGTGTAGATAG-3' |
| ADAMTS4 (J. Wang et al., 2021) | F: 5′-GAGGAGGAGATCGTGTTTCCA-3′ |
|  | R: 5′-CCAGCTCTAGTAGCAGCGTC-3′ |
| CCR2 (Nowak et al., 2023) | F: 5′-AGCCACAAGCTGAACAGAGA-3' |
|  | R: 5′-TGGTGACTTCTTCACCGCTC-3' |
| MCP1 (Henrioux et al., 2023) | F: 5′-ATAGCAGCCACCTTCATTCCC-3' |
|  | R: 5′-ATCTCCTTGGCCACAATGGTC-3' |
| IL1-ra (Sun et al., 2006) | F: 5'-GGCACTTGGAGACTTGTATGAAAGAT-3' |
|  | R: 5'-TCGCTGAGTACCTGCCAAGA-3' |
| NTRK1 | F: 5'-TCTTCAATGGCTCCGTGCTCAATG-3' |
|  | R: 5'-GGTCTCCAGATGTGCTGTTAGTGTC-3' |
| CGRP (Takano et al., 2017) | F: 5'-TTGCCCAGAAGAGAGCCTGTG-3' |
|  | R: 5'-TTGTTCTTCACCACACCCCCTG-3' |
| COX2 (Di et al., 2018) | F: 5’-CTGGCGCTCAGCCATACAG-3’ |
|  | R: 5’-CGCACTTATACTGGTCAAATCCC-3’ |
| TRPV4 (Q. Wang et al., 2024) | F: 5′-GCAGTTGGTCTGGTCCTCATTG-3′ |
|  | R: 5′-GTCCGGGTTCGAGTTCTTG-3′ |
| PIEZO1 (Schröder et al., 2023) | F: 5'-ACCTGCGTCATCATCGTGTG-3' |
|  | R: 5'-AGTTGGTGCTGTTGGGGAAG-3' |
| GAPDH (Takano et al., 2017) | F: 5'-TGTTGCCATCAATGACCCCTT-3' |
|  | R: 5'-CTCCACGACGTACTCAGCG-3' |

Note: The thermal cycling program included a 30 s hot start at 95 °C, followed by 40 cycles of denaturation (95 °C for 10 s) and annealing/extension (60 °C for 30 s). The melt curve analysis protocol comprised a 30 s hold at 95 °C, a 30 s stabilization at 65 °C, and a final dissociation ramp from 65 °C to 95 °C (0.5 °C/step, 5 s hold).

**References**

Di, F., Liu, J., Li, S., Yao, G., Hong, Y., Chen, Z.-J., . . . Du, Y. (2018). ATF4 contributes to ovulation via regulating COX2/PGE2 expression: a potential role of ATF4 in PCOS. *Frontiers in Endocrinology, 9*, 669. doi:10.3389/fendo.2018.00669

Henrioux, F., Navel, V., Belville, C., Charnay, C., Antoine, A., Chiambaretta, F., . . . Blanchon, L. (2023). Inflammation of dry eye syndrome: a cellular study of the epithelial and macrophagic involvement of NFAT5 and RAGE. *International Journal of Molecular Sciences, 24*(13), 11052. doi:10.3390/ijms241311052

Koelling, S., Clauditz, T. S., Kaste, M., & Miosge, N. (2006). Cartilage oligomeric matrix protein is involved in human limb development and in the pathogenesis of osteoarthritis. *Arthritis research & therapy, 8*, 1-10. doi:10.1186/ar1922

Mak, C. C., To, K., Fekir, K., Brooks, R. A., & Khan, W. S. (2022). Infrapatellar fat pad adipose-derived stem cells co-cultured with articular chondrocytes from osteoarthritis patients exhibit increased chondrogenic gene expression. *Cell Communication and Signaling, 20*(1), 17. doi:10.1186/s12964-021-00815-x

Neybecker, P., Henrionnet, C., Pape, E., Mainard, D., Galois, L., Loeuille, D., . . . Pinzano, A. (2018). In vitro and in vivo potentialities for cartilage repair from human advanced knee osteoarthritis synovial fluid-derived mesenchymal stem cells. *Stem cell research & therapy, 9*, 1-15. doi:10.1186/s13287-018-1071-2

Nowak, R., Trzeciak-Ryczek, A., Ciechanowicz, A., Brodkiewicz, A., Urasińska, E., & Kostrzewa-Nowak, D. (2023). The impact of different types of physical effort on the expression of selected chemokine and interleukin receptor genes in peripheral blood cells. *Cells, 12*(8), 1119. doi:10.3390/cells12081119

Schröder, A., Neher, K., Krenmayr, B., Paddenberg, E., Spanier, G., Proff, P., & Kirschneck, C. (2023). Impact of PIEZO1‐channel on inflammation and osteoclastogenesis mediated via periodontal ligament fibroblasts during mechanical loading. *European Journal of Oral Sciences, 131*(1), e12913. doi:10.1111/eos.12913

Shi, S., Wang, C., Chan, A., Kirmani, K., Eckert, G. J., & Trippel, S. B. (2019). Comparative effectiveness of structural versus regulatory protein gene transfer on articular chondrocyte matrix gene expression. *Cartilage, 10*(1), 102-110. doi:10.1177/1947603517719317

Sun, C. C., Su Pang, J. H., Cheng, C. Y., Cheng, H. F., Lee, Y. S., Ku, W. C., . . . Yang, C. M. (2006). Interleukin‐1 receptor antagonist (IL‐1RA) prevents apoptosis in ex vivo expansion of human limbal epithelial cells cultivated on human amniotic membrane. *Stem Cells, 24*(9), 2130-2139. doi:10.1634/stemcells.2005-0590

Takano, S., Uchida, K., Inoue, G., Minatani, A., Miyagi, M., Aikawa, J., . . . Takaso, M. (2017). Increase and regulation of synovial calcitonin gene-related peptide expression in patients with painful knee osteoarthritis. *Journal of pain research, 11*, 1099-1104. doi:10.2147/JPR.S135939

Wang, J., Wang, X., Ding, X., Huang, T., Song, D., & Tao, H. (2021). EZH2 is associated with cartilage degeneration in osteoarthritis by promoting SDC1 expression via histone methylation of the microRNA-138 promoter. *Laboratory Investigation, 101*(5), 600-611. doi:10.1038/s41374-021-00532-6

Wang, Q., Ji, C., Ali, A., Ding, I., Wang, Y., & McCulloch, C. A. (2024). TRPV4 mediates IL‐1‐induced Ca2+ signaling, ERK activation and MMP expression. *The FASEB Journal, 38*(11), e23731. doi:10.1096/fj.202400031R

Zuncheddu, D., Della Bella, E., Petta, D., Bärtschi, C., Häckel, S., Deml, M. C., . . . Basoli, V. (2022). Effect of glucose depletion and fructose administration during chondrogenic commitment in human bone marrow-derived stem cells. *Stem cell research & therapy, 13*(1), 533. doi:10.1186/s13287-022-03214-2

**Table S2.** Model fit statistics

|  | k = 4 | k = 5 | k = 6 | k = 7 |
| --- | --- | --- | --- | --- |
| Log-Likelihood | -643.363 | -658.062 | -591.203 | -519.311 |
| AIC | 1404.726 | 1524.124 | 1516.406 | 1540.622 |
| BIC | 1570.163 | 1815.742 | 1984.678 | 2244.431 |
| Silhouette Coefficient | 0.338 | 0.236 | 0.196 | 0.212 |

**Table S3.** Result of LEfSe analysis

| **Biomarker** | **Groups** | **LDA_value** | **p-value** |
| --- | --- | --- | --- |
| k__Bacteria.p__Bacillota.c__Bacilli.o__Lactobacillales.f__Streptococcaceae.g__Streptococcus.s__Streptococcus_agalactiae | Cluster 5 | 3.203 | 0.005 |
| k__Bacteria.p__Actinomycetota.c__Actinomycetia.o__Actinomycetales.f__Actinomycetaceae.g__Flaviflexus.s__Flaviflexus_salsibiostraticola | Cluster 4 | 3.089 | 0.003 |
| k__Bacteria.p__Campylobacterota.c__Campylobacteria.o__Campylobacterales.f__Arcobacteraceae.g__Aliarcobacter.s__Aliarcobacter_butzleri | Cluster 4 | 3.641 | 0.000 |
| k__Bacteria.p__Pseudomonadota.c__Gammaproteobacteria.o__Enterobacterales.f__Enterobacteriaceae.g__Providencia.s__Providencia_rettgeri_D | Cluster 4 | 3.507 | 0.004 |
| k__Bacteria.p__Pseudomonadota.c__Gammaproteobacteria.o__Pseudomonadales.f__Pseudomonadaceae.g__Pseudomonas_E.s__Pseudomonas_E_pudica | Cluster 4 | 3.008 | 0.029 |
| k__Bacteria.p__Pseudomonadota.c__Gammaproteobacteria.o__Pseudomonadales.f__Pseudomonadaceae.g__Stutzerimonas.s__Stutzerimonas_nitrititolerans | Cluster 4 | 3.406 | 0.004 |
| k__Bacteria.p__Actinomycetota.c__Actinomycetia.o__Actinomycetales.f__Actinomycetaceae.g__Flaviflexus | Cluster 4 | 3.077 | 0.003 |
| k__Bacteria.p__Campylobacterota.c__Campylobacteria.o__Campylobacterales.f__Arcobacteraceae | Cluster 4 | 3.653 | 0.000 |
| k__Bacteria.p__Campylobacterota.c__Campylobacteria.o__Campylobacterales.f__Arcobacteraceae.g__Aliarcobacter | Cluster 4 | 3.649 | 0.000 |
| k__Bacteria.p__Pseudomonadota.c__Gammaproteobacteria.o__Enterobacterales.f__Enterobacteriaceae.g__Providencia | Cluster 4 | 3.533 | 0.044 |
| k__Bacteria.p__Pseudomonadota.c__Gammaproteobacteria.o__Enterobacterales_A | Cluster 4 | 3.115 | 0.044 |
| k__Bacteria.p__Pseudomonadota.c__Gammaproteobacteria.o__Pseudomonadales.f__Pseudomonadaceae.g__Pseudomonas | Cluster 4 | 3.887 | 0.035 |
| k__Bacteria.p__Actinomycetota.c__Actinomycetia.o__Mycobacteriales | Cluster 3 | 3.419 | 0.045 |
| k__Bacteria.p__Pseudomonadota.c__Alphaproteobacteria.o__Rhizobiales.f__Rhizobiaceae.g__Phyllobacterium.s__Phyllobacterium_calauticae | Cluster 2 | 3.328 | 0.036 |
| k__Bacteria.p__Pseudomonadota.c__Gammaproteobacteria.o__Pseudomonadales.f__Moraxellaceae.g__Acinetobacter.s__Acinetobacter_johnsonii | Cluster 2 | 4.480 | 0.013 |
| k__Bacteria.p__Pseudomonadota.c__Alphaproteobacteria.o__Rhizobiales.f__Rhizobiaceae.g__Phyllobacterium | Cluster 2 | 3.334 | 0.036 |
| k__Bacteria.p__Pseudomonadota.c__Gammaproteobacteria.o__Burkholderiales.f__Burkholderiaceae_B | Cluster 1 | 4.374 | 0.022 |
| k__Bacteria.p__Pseudomonadota.c__Gammaproteobacteria.o__Pseudomonadales.f__Pseudomonadaceae | Cluster 1 | 4.406 | 0.015 |
| k__Bacteria.p__Pseudomonadota.c__Gammaproteobacteria.o__Pseudomonadales.f__Pseudomonadaceae.g__Stutzerimonas | Cluster 1 | 3.967 | 0.014 |

**Table S4.** Significant KEGG L3 pathways among clusters

| **Pathway** | **KW_p** |
| --- | --- |
| 2-Oxocarboxylic acid metabolism | 0.028 |
| AGE-RAGE signaling pathway in diabetic complications | 0.000 |
| Adipocytokine signaling pathway | 0.025 |
| Aminoacyl-tRNA biosynthesis | 0.017 |
| Amoebiasis | 0.026 |
| Arginine biosynthesis | 0.049 |
| Biosynthesis of vancomycin group antibiotics | 0.045 |
| Cell adhesion molecules | 0.000 |
| Cell cycle - Caulobacter | 0.023 |
| Chloroalkane and chloroalkene degradation | 0.014 |
| Dilated cardiomyopathy | 0.047 |
| ECM-receptor interaction | 0.001 |
| Ferroptosis | 0.016 |
| Glucagon signaling pathway | 0.041 |
| Glucosinolate biosynthesis | 0.014 |
| Human papillomavirus infection | 0.041 |
| Lipoic acid metabolism | 0.017 |
| Lysine degradation | 0.039 |
| Malaria | 0.001 |
| Naphthalene degradation | 0.009 |
| Nucleotide excision repair | 0.019 |
| Pantothenate and CoA biosynthesis | 0.046 |
| Protein export | 0.029 |
| Quorum sensing | 0.030 |
| RNA polymerase | 0.016 |
| Retinol metabolism | 0.006 |
| Type I polyketide structures | 0.003 |
| Viral carcinogenesis | 0.041 |

**Table S5.** Cluster-specific KEGG L3 pathways

| **Pathwway** | **Cluster** | **MW_p** | **MW_p_corr** |
| --- | --- | --- | --- |
| AGE-RAGE signaling pathway in diabetic complications | Cluster 3 | 0.000 | 0.000 |
| Cell adhesion molecules | Cluster 3 | 0.000 | 0.001 |
| Cell cycle - Caulobacter | Cluster 5 | 0.002 | 0.038 |
| ECM-receptor interaction | Cluster 3 | 0.000 | 0.001 |
| Ferroptosis | Cluster 5 | 0.002 | 0.038 |
| Lipoic acid metabolism | Cluster 5 | 0.001 | 0.027 |
| Malaria | Cluster 3 | 0.000 | 0.001 |
| Protein export | Cluster 5 | 0.003 | 0.050 |
| RNA polymerase | Cluster 5 | 0.002 | 0.038 |
| Retinol metabolism | Cluster 5 | 0.002 | 0.038 |
| Type I polyketide structures | Cluster 3 | 0.000 | 0.001 |

**Table S6.** The p-values for the taxon-gene pairs (|ρ| ≥ 0.2)

| Taxonomy | Gene | ρ | p | q |
| --- | --- | --- | --- | --- |
| s_Stutzerimonas_nitrititolerans | NTRK1 | 0.286 | 0.001 | 0.003 |
| s_Streptococcus_agalactiae | NTRK1 | 0.251 | 0.005 | 0.008 |
| s_Pseudomonas_E_pudica | NTRK1 | 0.272 | 0.002 | 0.005 |
| s_Providencia_rettgeri_D | NTRK1 | 0.344 | 0.000 | 0.001 |
| s_Phyllobacterium_calauticae | NTRK1 | 0.288 | 0.001 | 0.003 |
| s_Flaviflexus_salsibiostraticola | NTRK1 | 0.303 | 0.001 | 0.002 |
| s_Aliarcobacter_butzleri | NTRK1 | 0.344 | 0.000 | 0.001 |
| s_Acinetobacter_johnsonii | ADAMTS4 | 0.259 | 0.004 | 0.007 |
| s_Acinetobacter_johnsonii | CGRP | 0.268 | 0.003 | 0.005 |
| s_Acinetobacter_johnsonii | COL1A1 | -0.324 | 0.000 | 0.001 |
| o_Mycobacteriales | CCR2 | -0.251 | 0.005 | 0.008 |
| g_Providencia | NTRK1 | 0.344 | 0.000 | 0.001 |
| g_Phyllobacterium | NTRK1 | 0.288 | 0.001 | 0.003 |
| g_Flaviflexus | NTRK1 | 0.303 | 0.001 | 0.002 |
| g_Aliarcobacter | NTRK1 | 0.344 | 0.000 | 0.001 |
| f_Pseudomonadaceae | CGRP | -0.241 | 0.008 | 0.011 |
| f_Pseudomonadaceae | COL1A1 | 0.291 | 0.001 | 0.003 |
| f_Arcobacteraceae | NTRK1 | 0.344 | 0.000 | 0.001 |

**Table S7.** The p-values for the taxon-gene pairs (|ρ| ≥ 0.3)

| Taxonomy | Gene | ρ | p | q |
| --- | --- | --- | --- | --- |
| s_Providencia_rettgeri_D | NTRK1 | 0.344 | 0.000 | 0.000 |
| s_Flaviflexus_salsibiostraticola | NTRK1 | 0.303 | 0.001 | 0.001 |
| s_Aliarcobacter_butzleri | NTRK1 | 0.344 | 0.000 | 0.000 |
| s_Acinetobacter_johnsonii | COL1A1 | -0.324 | 0.000 | 0.000 |
| g_Providencia | NTRK1 | 0.344 | 0.000 | 0.000 |
| g_Flaviflexus | NTRK1 | 0.303 | 0.001 | 0.001 |
| g_Aliarcobacter | NTRK1 | 0.344 | 0.000 | 0.000 |
| f_Arcobacteraceae | NTRK1 | 0.344 | 0.000 | 0.000 |
